# Evaluation of Clinical and Radiographic Findings among Patients with Traumatic Dental Injuries Seeking Delayed Treatment

**DOI:** 10.1155/2021/9549508

**Published:** 2021-08-23

**Authors:** Sanjeeb Chaudhary, Harender Singh, Archana Gharti, Bhawana Adhikari

**Affiliations:** ^1^Department of Conservative Dentistry and Endodontics, School of Dental Sciences, Chitwan Medical College, Bharatpur-10, Chitwan, Nepal; ^2^Department of Public Health Dentistry, School of Dental Sciences, Chitwan Medical College, Bharatpur-10, Chitwan, Nepal; ^3^Department of Conservative Dentistry and Endodontics, People's Dental College and Hospital, Kathmandu, Nepal; ^4^Department of Conservative Dentistry and Endodontics, KIST Medical College, Lalitpur, Nepal

## Abstract

**Background:**

Time elapsed between trauma and treatment greatly influences the prognosis of traumatic dental injuries (TDIs). The aim of this study was to analyze clinical and radiographic findings related to complications of TDIs among patients seeking delayed treatment of such injuries.

**Materials and Methods:**

123 permanent teeth with a history of previous TDIs were included in the study. Clinical findings analyzed were the type of fracture, type and number of injured teeth, crown discoloration, and pulpal status of the injured tooth (pulpal diagnosis). The radiographic findings analyzed included pulp canal obliteration (PCO), root resorption (RR), and periapical radiolucency (PR). Statistical analysis included descriptive analysis.

**Results:**

Tooth discoloration was the most common presenting complaint (53.65%), while fall (48.78%) was the most frequent cause of trauma. The range of time duration between trauma and presentation for treatment was 5 months to 30 years (average time 12.82 years). Pulp necrosis (PN) was the most common complication (90.24%). Almost half of the teeth with PN had fracture injury and discoloration along with a high frequency of PR (78.37%). Even teeth with a normal appearance were found to have a high incidence of PN (76.92%) and PR (53.84%). The crown discoloration was the second most frequent finding (48.78%). Many teeth (41.66%) with vital pulp were also discolored. Most of the teeth (79.31%) with yellowish discoloration and all teeth with brownish discoloration were nonvital. A high frequency of PN (90%) and PR (78.33%) was found in teeth with discoloration. PR was the most common radiographic finding (69.10%), while PCO and RR were observed in 17.88% and 21.13% of teeth, respectively.

**Conclusion:**

The findings of this study support the fact that delayed treatment of TDIs leads to increased complications. PN was the most common complication followed by tooth discoloration, RR, and PCO among patients seeking delayed treatment after TDIs.

## 1. Introduction

Traumatic dental injuries (TDIs) are one of the most common causes of tooth loss and hence are a well-known public dental health problem. The main objective of the treatment of TDIs is the healing of the dental pulp and periradicular tissues [1].

Time elapsed between trauma and treatment is an important factor that greatly influences the prognosis of TDIs [2–5]. Similarly, treatment options and outcomes of treatment of traumatized teeth are significantly affected by the time duration between injury and the start of treatment. Treatment provided immediately after trauma has a significant effect on the prognosis of an injured tooth [5–7]. Timely treatment of an injured tooth is important for ensuring a favorable outcome and improving prognosis [5, 8, 9]. Immediate treatment of the traumatized tooth is the most important factor for preserving pulp vitality and for reducing complications [3, 6, 10].

Delayed treatment of TDIs has an unfavorable effect on the prognosis of injured teeth, which can cause loss of pulp vitality and thus poor prognosis [6, 8, 10, 11]. Treatment of TDIs is always aimed towards the reduction of complications. Delayed treatment is one of the factors linked with an increased rate of complications in teeth with TDIs [12, 13]. Delayed treatment of a traumatized tooth increases the risk of complications [5, 8, 11]. Even uncomplicated crown fractures can lead to various complications as a result of delayed treatment [3, 14].

However, treatment of TDIs is frequently neglected or delayed, which leads to various complications [5, 8]. Treatment of TDIs is time-consuming and expensive, and most of these injuries remain asymptomatic for a long time. Therefore, they are frequently ignored and left untreated [15, 16]. As a result, a low rate of treatment of TDIs is observed worldwide [4, 16]. Patients with TDIs come for treatment only after the appearance of symptoms or complications [5]. The development of these complications frequently leads to late presentation for treatment [13].

Although healing of dental pulp and adjacent tissue is the most favorable outcome of TDIs, complications such as pulp necrosis (PN), infection of the root canal system, pulp canal obliteration (PCO), crown discoloration, root resorption (RR), bone resorption, and ankylosis have frequently been associated with TDIs [1, 4]. These pulpal and periodontal complications related to TDIs can present early or even after several months or years of injury [3]. Recently, the use of magnetic resonance imaging (MRI) has been found to have an important application in the diagnosis, treatment, prognosis, and follow-up of these periapical bone lesions [17].

Therefore, the aim of this study was to analyze clinical and radiographic findings related to complications of TDIs among patients seeking delayed treatment of such injuries.

## 2. Materials and Methods

This study was conducted at the Department of Conservative Dentistry and Endodontics, School of Dental Sciences, Chitwan Medical College, Nepal, between January 2018 and December 2019, for a period of two years. It included patients coming for delayed treatment of teeth with a previous history of TDIs to permanent dentition. Informed consent was taken according to ethical principles. This study was approved by the Institutional Review Board of the Chitwan Medical College (Ethical Clearance No.: CMC-IRC/076/077–148).

A total of 123 permanent teeth with a previous history of TDIs were evaluated in this study. The study included 82 patients comprising 47 males (57.31%) and 35 females (42.68%). The ratio between male and female is 1.34 : 1. The age of the patient ranged from 14 to 53 years (mean age was 28.03 years).

A thorough clinical and radiographic examination was conducted using a digital intraoral periapical radiograph (Carestream Health Inc) exposed with a paralleling technique and an exposure time 0.125 seconds at 60 kV and 7 mA. The patient's name, age, gender, chief complaint (reason for seeking treatment), time elapsed between trauma and presentation for treatment, and the etiology of trauma were investigated.

Findings from the clinical examination and tests, as well as radiographic examination, were documented. The clinical findings analyzed were type of fracture injury, type and number of injured teeth, crown discoloration, intraoral sinus, swelling, and pulpal status of the injured tooth (pulpal diagnosis). The radiographic findings analyzed were PCO, RR, and periapical radiolucency (PR).

The diagnosis of PN was made based on clinical and radiological findings. The tooth was diagnosed as PN when clinical findings (pain, swelling, crown discoloration, negative response to EPT, tenderness to percussion, and presence of sinus) were observed along with radiographic findings (PR and or RR) [18]. PCO and RR were diagnosed based on radiographic analysis. Diagnosis of PCO and RR was based on radiographic analysis. Radiographic analysis was done separately by two endodontists using the RadioVisioGraphy (RVG) imaging system, Dental Imaging Software, 6.14.4 (Carestream Health Inc. 2014).

Teeth with a previous history of treatment were excluded from the study. Similarly, teeth without a previous history of TDIs were also excluded from the study.

The data collected were subjected to descriptive analysis and chi-square tests were applied for association analysis. Statistical analysis of the obtained data was performed using Statistical Package for the Social Sciences (SPSS, version 22.0 (IBM SPSS)). The level of significance was set at 5%.

## 3. Results

The most frequently observed history of TDIs was in the age group 14–20, followed by the age group 21–25. Comparison of the frequency of TDIs between genders and age groups found no significant difference (*p* > 0.05). The distribution of TDIs with respect to age groups, gender, and etiology is shown in Table 1. Fall (48.78%) was the most frequent cause of trauma, followed by impact injury (20.04%). Similarly, the comparison of the frequency of the causes between the genders and differences between the cause of trauma and age distribution was also not statistically significant (*p* > 0.05).

The most commonly traumatized tooth was the maxillary right central incisor (36.58%), followed by the maxillary left central incisor (23.57%). Maxillary teeth (74%) were more frequently involved than mandibular teeth (26%). The distribution of the affected tooth type is presented in Table 2. Tooth distribution, according to clinical and radiographic findings, is shown in Table 3. The most common presenting complaint was tooth discoloration (44; 53.65%) followed by swelling (22; 26.82%), pus discharge (14; 17.07%), and pain (2; 2.43%). The swelling was found in association to 14 teeth, whereas intraoral sinus was seen in relation to 13 teeth. Similarly, swelling associated with intraoral sinus was observed in 5 teeth.

The range of time duration between trauma and presentation for treatment was 5 months to 30 years. The average time of presentation after trauma was 12.82 years (within a year = 3 (3.65%), 2–4 years = 16 (19.51%), 5–7 years = 13 (15.85%), 8–10 years = 15 (18.29%), 11–15 years = 21 (25.60%), 16–20 years = 6 (7.31%), 21–25 years = 4 (4.87%), and 26–30 years = 4 (4.87%)).

Vital pulp was detected in (9.75%) teeth upon diagnosis. Among them, 33.33% of teeth had a fracture, whereas 41.66% of teeth had discoloration. Similarly, on radiographic examination and 50% of teeth had PCO, and 16.66% of teeth had RR. Similarly, nonvital pulp (PN) was diagnosed in 90.24% of teeth. Among them, 44.14% of teeth had a fracture, and 49.54% of teeth were discolored. Likewise, 78.37% of teeth were associated with PR, 13.51% of teeth had PCO, and 20.72% had RR on radiographic evaluation. The tooth distribution, clinical and radiographic findings associated with pulpal diagnosis are shown in Table 4.

Likewise, on clinical examination, 21.13% of teeth had a normal appearance (NA). Among them, 76.92% of teeth were diagnosed as having PN. 53.84% of teeth were related to PR, 38.46% of teeth had PCO, and 19.23% had RR on radiographic findings. Enamel fracture (E#) was seen in 8.13% of teeth, of which 90% of teeth had PN. PR was found in 90% of teeth, while 10% had PCO and 50% had RR. Enamel dentin fracture (ED#) was observed in 26.01% of teeth. Among them, 84.37% teeth were diagnosed to have PN. Also 75% teeth had PR, and 28.12% of teeth were associated with RR. Similarly, all teeth with complicated crown fractures were diagnosed with PN. Among them, 72.72% of teeth were linked to PR on radiographic examination.

Tooth discoloration was a frequent finding (48.78%) in this study. Among them, 48.33% of teeth had yellowish discoloration. 79.31% of teeth with yellowish discoloration had PN. Also, 68.96% of teeth were associated with PR and 27.58% of teeth had PCO and 31.03% had RR. All teeth with brownish discoloration (51.66%) were nonvital. 87.09% of teeth with brownish discoloration had PR, while only 3.22% of them had PCO and RR 16.12%. Overall, a high incidence of PN (54; 90%) and PR (78.33%) was found in teeth with discoloration. However, the frequency of RR (23.33%) and PCO (15%) observed was low in teeth with discoloration. The tooth distribution, pulpal diagnosis, and clinical and radiographic findings related to clinical examination are shown in Table 5.

Upon analysis of radiographs, 13% of teeth had normal radiographic findings (NRF). Among them (25%) were discolored, whereas (56.25%) had fracture injuries. Also, 50% of teeth with NRF were nonvital.

PR (69.10%) was the most common radiographic finding. 52.94% of teeth had discoloration, and 48.23% of teeth had fracture injury among the teeth with PR. All teeth with PR were nonvital, while 22.35% teeth had RR and 8.23% teeth had PCO.

PCO was detected in 17.88% of teeth. Among them, half were discolored. The yellowish discoloration was found in 72.72% of teeth, whereas only 27.27% of teeth had brownish discoloration. Also, 63.63% of teeth with PCO were nonvital. On radiographic examination, 31.81% of teeth were associated with PR and 27.27% of teeth had RR in teeth with PCO.

Likewise, 21.13% of teeth were found to have RR on radiographic evaluation. Among them, 88.46% of teeth had PN, 50% of teeth were discolored, while 73.07% of teeth had PR and 26.92% of teeth had PCO. The tooth distribution, pulpal diagnosis, and clinical and radiographic findings according to radiographic examination are shown in Table 6.

## 4. Discussion

Immediate treatment after dental trauma significantly improves the prognosis of the injured tooth and is equally important for preventing and reducing complications. Late presentation for treatment after TDIs was identified as the reason for higher frequency of complications in a recent study [13]. Timely treatment of TDIs also helps in better management of posttraumatic complications [19]. The time interval between injury and presentation for treatment ranged between 5 months and 30 years in this study.

Many patients with TDIs do not seek treatment as long as it remains asymptomatic [20]. Tooth discoloration (53.65%) was the most common presenting complaint, followed by swelling in this study. This indicates that several patients, unless esthetically concerned or presence of pain and discomfort, do not come for treatment of TDIs.

Patients generally seek immediate treatment for severe injuries due to road traffic accidents, assaults, or sports. However, traumas due to impact and fall injury are considered mild injuries and are often neglected since they are seldom severe or symptomatic. Hence, patients tend to delay or ignore treatment for these kinds of injuries. This may explain the findings of this study, where fall (48.78%) was the most frequent cause of trauma. Most of the TDIs were due to fall and impact injury (Table 1).

PN has been found to be the most frequent posttraumatic complication in all types of TDIs [5, 14, 21]. Depending on the severity of TDIs the prevalence of PN ranges from 17–100%. In a recent study, PN was the most common complication among patients with late presentation for treatment [13]. Similar results were found in the present study. PN was the most frequent complication, with 90.24% of teeth diagnosed with PN. Almost half of the teeth with PN had fracture injury and discoloration. Also, a high frequency of PR (78.37%) was seen in teeth with PN. However, the rate of PCO and RR was low (Table 4). Nevertheless, some studies have reported a lower frequency of PN. A similar prevalence of PN (26.9%) was reported in a study by Hecova et al. and Kallel et al., while Lin et al. reported a prevalence of PN at (34.2%) [5, 14, 21].

The prevalence of PN is very low in enamel-fractured and dentin-fractured teeth without an associated luxation injury. However, the risk of PN is higher in teeth with dentin fracture compared to teeth with enamel fracture [2, 3, 18, 22]. The incidence of PN in enamel and dentin-fractured teeth in a recent study was 4% and 22%, respectively [18]. Most of the complications were found in teeth with enamel-dentine fracture (93.4%), followed by concussion injury (55.6%) in another recent study among patients coming for late treatment of TDIs [13]. Although crown fracture has a low risk of PN when proper treatment is provided, PN as a result of crown fracture was reported by Robertson et al. to be more common [3]. A high rate of PN (88.67%) and PR (77.35%) was found in teeth with fracture injury. 90% teeth with E# and 84.37% teeth with ED# were nonvital, whereas all teeth with complicated crown fracture were nonvital in this study (Table 5). This suggests that even teeth with uncomplicated fractures can lead to a rise in complications due to delayed treatment.

PN in uncomplicated crown fractures is rare and ranges between 2% and 5%. However, PN is very common in fractured teeth without treatment. Since complications can arise from the invasion of bacteria into the dentinal tubules, an increased PN is found even in the absence of luxation injuries, particularly in teeth with delayed treatment [9, 22, 23].

PN was also a common finding in fractured teeth among patients seeking delayed treatment in another previous study [24]. Therefore, protection of the exposed dentin should be done as soon as possible. Similarly, immediate sealing of the exposed pulp is also essential for a favorable prognosis [7]. Even teeth with NA were found to have a high rate of PN (76.92%) and PR (53.84%) in this study. Similarly, half of the teeth with NRF were also nonvital.

PN, PCO, RR, and ankylosis are important complications linked to luxation injures. Luxation injuries due to the damage to the neurovascular bundle are associated with a higher incidence of PN [18]. Many studies have found PN to be the most common complication in teeth with luxation injury [8, 25]. The development of PN after luxation injuries is related to several significant clinical factors such as stage of root development, type, and extent of luxation injury, type of treatment, and delayed initial treatment [26–29]. Teeth with complete root development have a high risk of developing PN [14, 18, 26]. PN is especially more common in teeth with luxation injury having mature apex [30, 31]. In a retrospective study, teeth with completed root development demonstrated a higher prevalence of PN in all types of luxation injuries [14]. All of the teeth evaluated in this study had complete root development, which could be the reason for a high incidence of PN observed.

Concussion and subluxation injuries have a low risk of PN, whereas lateral luxation and intrusion have a high rate of PN [8, 14, 31–33]. Various studies have also found a high incidence of PN in teeth with concomitant fracture and luxation injury [3, 27–29]. A study found an increased risk of PN in crown fractured teeth with an associated concussion, subluxation, extrusion, lateral luxation, and intrusion injury in ascending order [18].

Time period of development of PN after dental trauma varies depending on the type of injury. Although most of the PN developed within 12 months of trauma, the incidence of PN within 3 months was significantly higher in a study [18]. Therefore, the first 3 months after TDIs are very important with respect to monitoring and follow-up [18]. Similarly, most of the PN was detected within the first six months after trauma in other studies [9, 22, 34]. However, it is agreed that PN mostly occurs within the first year after trauma [14, 21]. PN was observed as early as 5 months after trauma in the present study. Therefore, frequent follow up is essential for early detection and treatment of complications. Hence, a follow-up period of more than one year is recommended for the evaluation of pulpal healing.

PCO is a pulpal healing complication commonly found in teeth with luxation injuries, root fracture, and reimplanted teeth [13, 24, 35, 36]. The prevalence of PCO after TDIs has been found to range from 3.7% to 40% [21, 35, 37]. In this study, 17.88% of the teeth were diagnosed with PCO (Table 6). According to Bastos and Cortes, teeth with extrusive and lateral luxation injury demonstrate the highest frequency of PCO which is also supported by other studies [8, 30, 31, 35, 38]. The incidence of PCO is significantly associated with the degree of displacement and to the degree of root development [3, 30]. PCO is also considerably more frequent in teeth with incomplete root development [26]. PCO can be detected clinically 3 months after trauma [37].

Although very rare, PN can occur in teeth with PCO. PN is a late complication associated with PCO and ranges from 1% to 16% [35, 37]. However, a high frequency of PN (63.63%) was observed in teeth with PCO in the present study. Most of the teeth with PCO were nonvital. PN in teeth with PCO has been significantly related to the severity of the injury and the stage of root development [35]. All of the teeth with PN and PCO in this study had complete root development.

Teeth with PCO generally have discoloration, which is clinically seen as a yellowish discoloration of the crown [24]. Yellowish discoloration in teeth with PCO is a frequent finding ranging from 8.3% to 79%. In contrast, brownish discoloration is very rare (1%) in teeth with PCO [35]. A study detected yellowish discoloration in 67.4% and brownish discoloration in 12.3% of teeth with PCO. Similarly, 33.3% of teeth with PCO had periapical lesions, while 30.7% of teeth had NRF [39]. In this study, half of the teeth with PCO were discolored. Yellowish discoloration (72.72%) was more common than brownish discoloration (27.27%). However, the frequency of PR and RR was low (Table 6). Interestingly, PCO was found only in teeth with E# in this study. This suggests that teeth with mild trauma are more likely to develop PCO.

Tooth discoloration, a long-term complication of dental trauma, is a frequent finding in a traumatized tooth. Grayish and brownish discoloration of a traumatized tooth indicates PN, while yellowish discoloration is a sign associated with PCO. Patients with mild injuries such as concussion and subluxation usually have negligible symptoms and hence seek treatment only when it becomes symptomatic. These patients with discolored teeth often come for delayed treatment [24]. Tooth discoloration was the second most common complication observed in a study related to patient seeking delayed treatment after TDIs [13]. Similarly, crown discoloration was the second most common finding (48.78%) in this study. 48.33% teeth had yellowish discoloration, while 51.66% teeth were of brownish discoloration (Table 5).

Upon further evaluation, most of the teeth (79.31%) with yellowish discoloration were nonvital with a high incidence of PR (68.96%). A very high frequency of PN was also seen in teeth with brownish discoloration. All teeth with brownish discoloration were nonvital. Most of the teeth with brownish discoloration have been associated with periapical lesions [35, 39]. A high incidence of PR (87.09%) in teeth with brownish discoloration was also found in this study. Overall, a high frequency of PN (90%) and PR (78.33%) was observed in teeth with discoloration. In contrast, the rate of RR and PCO was low. The results of this study are in contrast to a former study in which PCO was found in 79.2%, and PN was found in 20.8% of discolored teeth [24]. This difference may be due to the inclusion of only discolored teeth in the previous study. Furthermore, the time duration of arrival for treatment was earlier (mean = 13.2 months) in the previous study, whereas in the present study, the time duration for arrival for treatment was late (mean = 12.82 years). Additionally, many teeth (41.66%) with vital pulp were also discolored in this study.

PR was the most common radiographic finding (69.10%) (Table 6). Almost half of the teeth with PR had fracture injury and discoloration. All teeth with PR were nonvital. However, the incidence of PCO and RR was low. A very low frequency of periodontal complications is found after concussion and subluxation injuries [25, 40]. In the same way, the risk of periodontal complications in teeth with extrusive luxation and lateral luxation injuries are also generally low [41].

RR is an important periodontal healing complication of TDIs. RR was observed in (21.13%) of teeth with a high rate of PN (88.46%) and PR (73.07%). Half of the teeth with RR were also discolored (Table 6).

Surprisingly, RR was not detected in teeth with a complicated crown fracture in this study since RR is mostly associated with luxation injury. RR was more frequently associated with intrusive luxation (93%), followed by avulsions (89%), lateral luxation (80%), and extrusive luxation (77%) in a recent study [42]. This is also supported by a recent review and various studies stating RR to be mostly associated with intrusive luxation, followed by extrusive luxation, lateral luxation, subluxation, and concussion injury [30, 32, 33, 43]. Early repositioning of luxated and displaced teeth helps to prevent the onset of RR [12]. The risk of RR is likewise high in teeth with concomitant fracture and luxation injury because of the increased risk of PN in such teeth. RR is generally seen within five months from injury [21, 22]. However, the use of cone-beam computed tomography (CBCT) has been found to be superior in the early detection and diagnosis of PR and RR [44].

Other periodontal complications related to TDIs such as marginal bone loss, mobility, transient apical breakdown, and cervical-invasive resorption, however, were not evaluated in this study.

Early diagnosis of complications is very important since management of such tooth is difficult. Therefore, timely treatment and follow-up of an injured tooth, according to guidelines, are important for ensuring a favorable outcome, protecting pulp vitality, and reducing complications [34]. Similarly, the concept of torque range as proposed by a recent study can be very useful for the prevention of intracanal separation of the instrument and other iatrogenic errors during root canal preparation of a tooth with PCO [45].

Knowledge about the various consequences in teeth with TDIs can help to provide better treatment and reduce complications. Likewise, information regarding the timeline of the development of possible complications of TDIs is also essential to anticipate and prevent them.

A traumatized tooth can have multiple signs and symptoms related to complications. Information from this study may help to predict the possibility of various likely complications related to TDIs and identify these complications early. Outcomes of this study may additionally assist in monitoring and follow-up of such teeth as well. Future studies related to the management of complications of TDIs with follow-up of the patients can be conducted, which might be valuable in better understanding of these complications linked to TDIs.

## 5. Conclusion

The findings of this study support the fact that delayed treatment of TDIs leads to increased complications. Pulp necrosis was the most common complication followed by tooth discoloration, root resorption, and pulp canal calcification among patients seeking delayed treatment after TDIs in this study.

## Figures and Tables

**Table 1:** Distribution of age, gender, and etiology.

Age distribution	No. (%)	M : F (no.)	Fall: impact: RTA: sports: assault	(*p*)
14–20	20 (24.39%)	12 : 8	12 : 5 : 3 : 0 : 0	*p* > 0.05
21–25	19 (23.17%)	11 : 8	10 : 4 : 2 : 2 : 1	
26–30	17 (20.73%)	11 : 6	6 : 7 : 1 : 3 : 0	
31–35	8 (9.75%)	4 : 4	5 : 3 : 0 : 0 : 0	
36–40	8 (9.75%)	4 : 4	2 : 1 : 4 : 1 : 0	
41–45	5 (6.09%)	2 : 3	3 : 2 : 0 : 0 : 0	
46–50	3 (3.65%)	2 : 1	2 : 1 : 0 : 0 : 0	
51–55	2 (2.4%)	1 : 1	0 : 0 : 2 : 0 : 0	
Total	82	47 : 35	40 : 23 : 12 : 6 : 1	

**Table 2:** Distribution of dental trauma according to affected tooth type.

Type of injured tooth: number								
Incisors (120)	11 = 45	12 = 11	21 = 29	22 = 4	31 = 9	32 = 7	41 = 10	42 = 5
Canines (3)	13 = 1	23 = 1	33 = nil	43 = 1				

Teeth numbering, according to the FDI numbering system.

**Table 3:** Tooth distribution according to clinical and radiographic findings.

Tooth	13	12	11	21	22	23	32	31	41	42	43	Total
Vital pulp			5	6	1							12
Pulp necrosis	1	11	40	23	3	1	7	9	10	5	1	111
Normal appearance (NA)		1	7	2	2		4	3	2	4	1	26
Enamel fracture (E#)			6	2	1				1			10
Enamel dentin fracture (ED#)	1	5	15	8		1		2				32
Complicated crown fracture (EDP#)		2	4	1			2	2				11
Brownish discoloration (BD)		1	13	11				3	2	1		31
Yellowish discoloration (YD)	1	2	14	8		1	1		2			29
Normal radiographic findings (NRF)		1	6	5	1		1	1		1		16
Periapical radiolucency (PR)	1	9	27	19	2	1	5	7	9	4	1	85
Pulp canal obliteration (PCO)		1	11	6	2					2		22
Root resorption (RR)		2	13	7	3				1			26

**Table 4:** Distribution of pulpal diagnosis.

Diagnosis	Patient	Teeth	Clinical findings	Radiographic findings
Vital pulp	9	12 (9.75%)	(NA = 4, # = 3, *D* = 4, # + *D* = 1)	NRF = 6, PCO = 4, PCO + RR = 2
Pulp necrosis	73	111 (90.24%)	(NA = 29, # = 27, *D* = 33, #+*D* = 22)	NRF = 14, PR = 64, PCO = 5, RR = 2, PR + PCO = 5, PR + RR = 16, PCO + RR = 3, PR + PCO + RR = 2

# = crown fracture; *D* = crown discoloration.

**Table 5:** Distribution of clinical findings.

Clinical findings	Number of patients	Number of teeth	Clinical findings	Diagnosis	Radiographic findings
Normal appearance	21	26 (21.13%)	Normal appearance	(NP = 6; 23.07%, PN = 20; 76.92%)	NRF = 3, PR = 12, PCO = 5, PR + PCO = 1, PCO + RR = 4, PR + RR = 1
E#	10	10 (8.13%)	(E# = 5, E#+*D* = 5)	(NP = 1; 10%, PN = 9; 90%)	NRF = 1, PR = 4, PR + RR = 4, PR + PCO + RR = 1
ED#	25	32 (26.01%)	(ED# = 16, ED#+*D* = 16)	(NP = 5; 15.62%, PN = 27; 84.37%)	NRF = 7, PR = 16, PR + RR = 8, RR = 1
EDP#	9	11 (8.94%)	(EDP# = 9, EDP#+*D* = 2)	(PN = 11; 100%)	NRF = 3, PR = 8
BD	27	31 (51.66%)	(*D* = 19, ED#+*D* = 10, EDP#+*D* = 2)	(PN = 31; 100%)	NRF = 3, PR = 22, PR + PCO = 1, PR + RR = 4, RR = 1
YD	27	29 (48.33%)	(*D* = 18, E#+*D* = 5, ED#+*D* = 6)	(NP = 6; 20.68%, PN = 23; 79.31%)	NRF = 3, PR = 11, PCO = 4, PR + PCO = 2, PR + RR = 6, RR = 1, PCO + RR = 1, PR + PCO + RR = 1

**Table 6:** Distribution of radiographic findings.

	Number of patient	Number of teeth	Clinical findings	Diagnosis	Radiographic findings
Normal radiographic findings (NRF)	14	16 (13%)	(NA = 5, E# = 1, ED# = 4, EDP# = 2, *D* = 2, ED#+*D* = 2)	(NP = 8; 50%, PN = 8; 50%)	NRF
Periapical radiolucency (PR)	65	85 (69.10%)	(NA = 17, E# = 4, ED# = 12, EDP# = 7, *D* = 27, E#+*D* = 5, ED# + *D* = 12, EDP#+*D* = 1)	(PN = 85; 100%)	PR = 61, PR + PCO = 5, PR + RR = 17, PR + PCO + RR = 2
Pulp canal obliteration (PCO)	20	22 (17.88%)	(NA = 11, *D* = 10, E# + *D* = 1)	(NP = 8; 36.36%, PN = 14; 63.63%)	PCO = 10, PCO + PR = 6, PCO + RR = 5, PCO + PR + RR = 1
Root resorption (RR)	21	26 (21.13%)	(NA = 6, E# = 1, ED# = 6, E# + *D* = 4, ED# + *D* = 3, *D* = 6)	(NP = 3; 11.53%, PN = 23; 88.46%)	RR = 2, RR + PR = 17, RR + PCO = 5, RR + PR + PCO = 2

# = crown fracture; *D* = crown discoloration.

## Data Availability

The data used to support the findings of this study are available from the corresponding author upon request.
